# Alberta Stroke Program Early CT Score applied to hyperdense lesion on noncontrast CT immediately post-thrombectomy is a predictor of poor outcome in acute ischemic stroke: A case-control study

**DOI:** 10.1097/MD.0000000000030514

**Published:** 2022-09-09

**Authors:** Zhengzhou Yuan, Yuan Yang, Ying Luo, Xiu Chen, Hua Luo, Jinglun Li, Renliang Meng, Yang Xie, Li Jiang, Zhiyu Lv, Benbing Rong, Zuoxiao Li

**Affiliations:** a Department of Neurology, The First Affiliated Hospital of Chengdu Medical College, ChengDu, China; b Department of Neurology, Affiliated Hospital of Southwest Medical University, LuZhou, China.

**Keywords:** ASPECTS, hyperdense lesion, mechanical thrombectomy, prognosis, stroke

## Abstract

We aimed to evaluate whether Alberta Stroke Program Early CT Score (ASPECTS) applied to hyperdense lesion on noncontrast CT obtained immediately post-thrombectomy (post-ASPECTS) is useful for predicting poor outcome. We retrospectively reviewed patients who underwent noncontrast CT (NCCT) immediately after mechanical thrombectomy between January 2017 and July 2020 in our comprehensive stroke center. We collected baseline NCCT and post-ASPECTS score. The sensitivity, specificity, and positive and negative predictive values of the post-ASPECTS in predicting clinical outcome were calculated. A total of 223 patients were included. The hyperdense lesion on NCCT immediately after endovascular thrombectomy presented in 85.7% (191/223) patients, poor clinical outcome was in 56.1% (112/191) of hyperdense lesion patients. Low post-ASPECTS was associated with poor outcome (OR 0.390; 95% CI 0.258-0.589; *P* = .001), with an AUCROC curve of 0.753 (95% CI 0.684–0.822), while baseline NCCT-ASPECTS was not (OR 0. 754; 95% CI 0. 497-1.144; *P* = .185). A score ≤ 7 in post-ASPECTS was the best cut-off to poor clinical outcome (sensitivity 84.8%; specificity 52.7%; positive predictive value 68.4%; negative predictive value 73.8%). Our results point to the proportion of patients who present hyperdense lesion on NCCT is very high, post-ASPECTS could predict poor clinical outcomes in patients with stroke treated with endovascular mechanical thrombectomy, and post-ASPECTS may achieved better predictive value than baseline ASPECTS.

## 1. Introduction

Reperfusion therapy after endovascular thrombectomy (EVT) improves clinical outcome in patients with acute ischemic stroke (AIS) caused by anterior circulation large vessel occlusion.^[1–7]^ It is routine to detect hemorrhage and other complications by noncontrast computed tomography (NCCT) immediately post-thrombectomy, and hyperdense lesions on NCCT can be observed in 55% to 86% of patients immediately after EVT.^[8,9]^ The presence of a hyperdense lesion on NCCT predicts hemorrhagic transformations at 24 hours post-thrombectomy,^[10]^ and a massive hyperdense lesion predicts malignant brain edema and an unfavorable clinical outcome.^[11]^ However, a quantitative or semi-quantitative analysis of the relationship between patient’s clinical outcome and hyperdense lesions on NCCT obtained immediately after EVT has not been performed.

The Alberta Stroke Program Early CT Score (ASPECTS) grading system is an effective and convenient semi-quantitative method of estimating the topography of tissue changes in the brain with noncontrast CT during the acute phase. The American Heart Association guidelines recommended a baseline ASPECTS between 6 and 10 as 1 of the conditions for screening patients for EVT.^[12]^ Low admission ASPECTS have been shown to correlate with unfavorable functional outcomes.^[13]^ However, patients with suitable admission ASPECTS undergo EVT, and only half of them experience favorable clinical outcomes.^[14]^ Some patients have a high baseline ASPECTS and successful recanalization after EVT, but present a larger ischemic core on post-thrombectomy NCCT.

Compared with the initial National Institutes of Health Stroke Scale (NIHSS) score, the post-thrombectomy NIHSS score had better discrimination power for functional recovery at the 3 months follow-up.^[15]^ We hypothesize that ASPECTS applied to hyperdense lesions on NCCT obtained immediately after EVT may be a predictor of poor outcome in anterior circulation AIS. Because the ASPECTS is convenient and widely used, and using ASPECTS to semi-quantitatively assess hyperdense lesions on NCCT obtained immediately post-thrombectomy as a predictive factor has rarely been reported, we conducted a retrospective study to test our hypothesis.

## 2. Materials and methods

### 2.1. Patient selection and evaluation

We analyzed our prospectively collected database of all patients with AIS with anterior circulation large vessel occlusion who had undergone EVT by 3 primary operators at Affiliated Hospital of Southwest Medical University, Luzhou, Sichuan, China from January 2017 to July 2020. The ethics committee of the affiliated hospital of Southwest Medical University approved this study (No. 20200247). Investigations were conducted according to the principles expressed in the Declaration of Helsinki. We enrolled patients in the analysis if they underwent EVT due to large vessel occlusion of the internal cerebral artery or the main trunk of the middle cerebral within 6 hours, or within 6 to 24 hours of the last known normal who meet other DAWN eligibility criteria^[6]^; have a prestroke modified Rankin scale (mRS) score of 0 to 1; had undergone NCCT scanning of the brain within 0.5 hours after EVT; have a follow-up NCCT scan at 24 hours after EVT; and had been followed for longer than 90 days after the intervention. Patients were excluded if they had acute occlusion of large arteries in the posterior circulation; or lost non-contrast CT data or had unsatisfactory image quality caused by motion artifacts.

### 2.2. Protocol for EVT procedure and imaging

EVT procedures were carried out by 2 of a pool of 3 radiologists with 5 to 15 years of experience in interventional neuroradiology on an Allura Xper FD 20 X-ray system (Philips Healthcare, Best, Netherlands). All patients were treated according to the guidelines: using a stent retriever device, such as Trevo (Stryker Neurovascular, Mountain View, CA) or Solitaire FR (ev3, Irvine, CA) or Aperio (acandis, Pforzheim, Germany). Alternatively, an aspiration catheter, such as ACE (Penumbra, Alameda, CA) or AXS Catalyst 6 (Stryker Neurovascular, Mountain View, CA), if aspiration was unsuccessful followed by stent retriever device. NCCT examinations were performed on a LightSpeed 16 scanner system (GE, General Electric Company, Boston, MA) with an axial 5-mm section thickness. NCCT scans of the brain were taken within 0.5 hours after EVT in all patients. We guided the time of started anticoagulants by imaging and clinical symptoms at 24 hours after thrombectomy.

### 2.3. Definition and evaluation of a hyperdense lesion

**Figure 1.**
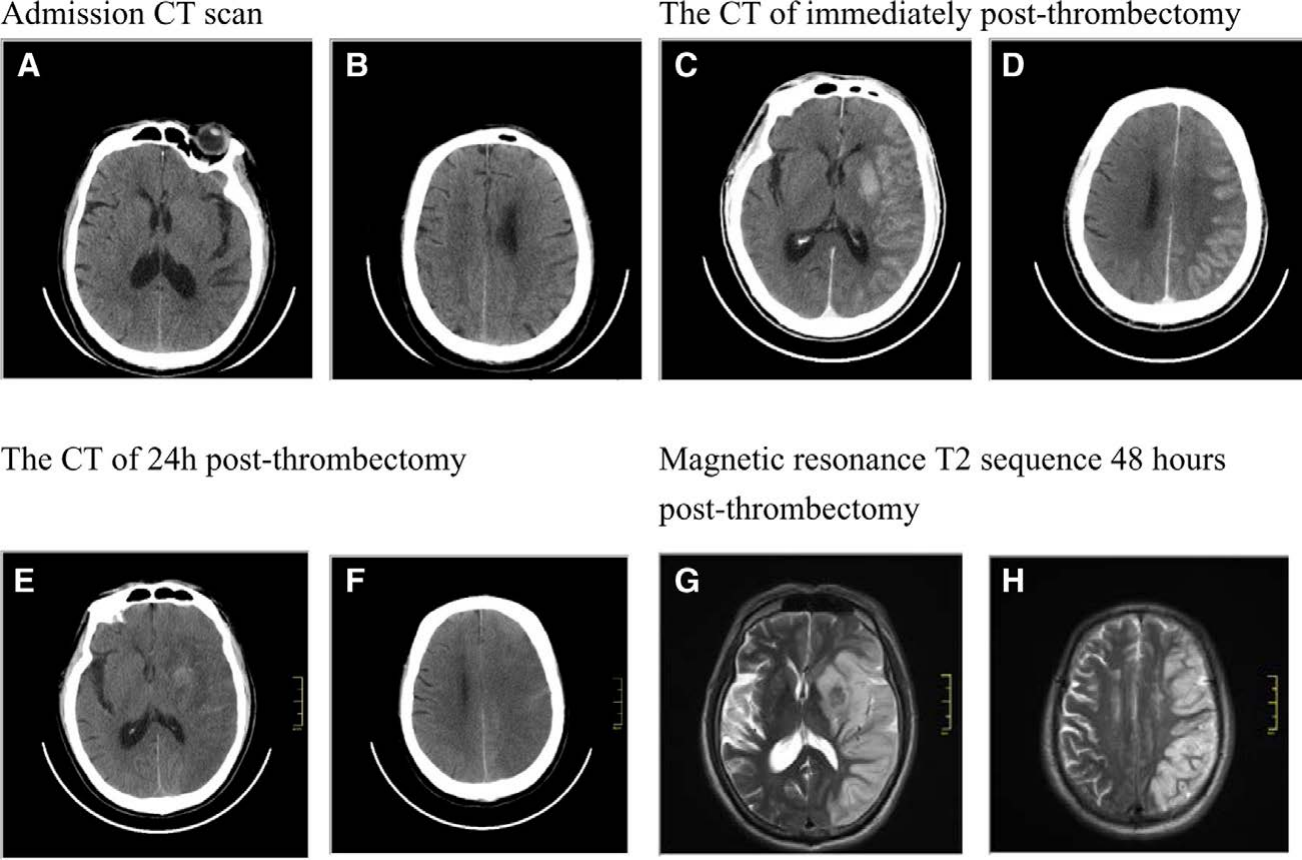
Example of a positive hyperdense lesion patient. The patient with baseline ASPECTS of 7, post-ASPECTS was 2 (caudate nucleus and posterior limb of the internal capsule were exempt), and mRS was 5 on 90 days followed-up. (A and B) Admission CT scan; (C and D) CT of immediately post-thrombectomy; (D and E) CT of 24 h post-thrombectomy; (F and G) magnetic resonance T2 sequence 48 hours post-thrombectomy. ASPECTS = Alberta Stroke Program Early CT Score, mRS = modified Rankin Scale.

According to previous studies,^[16,17]^ a hyperdense lesion was defined as a high density (>40 HU) image on the NCCT obtained immediately post-thrombectomy that conformed to the anatomic boundaries of normal structures, and had no surrounding edema, and a mass effect intracranial hematoma on NCCT was excluded. Two experienced neurologists (H.L. and X.C.) blinded to the clinical data, used the ASPECTS to assess the hyperdense lesion on NCCT immediately after EVT (Fig. 1), while the inconsistent data were reviewed by another experienced neurologist (Z.L.) and resolved through consensus discussion. ASPECTS evaluation of hyperdense lesions was calculated by subtracting 1 point subtracted for any hyperdense lesion in each defined region on the NCCT scan immediately after EVT, similar with assessed the admission baseline NCCT ASPECTS.

### 2.4. Data measurement

The following clinical data were extracted from the medical records: age, sex, diabetes mellitus, hypertension, atrial fibrillation, systolic blood pressure on admission, plasma glucose level, baseline ASPECTS (range, 0 to 10, with 1 point subtracted for any evidence of early ischemic change in each defined region on the CT scan), use of IV tissue plasminogen activator, pre-thrombectomy NIHSS score, procedural technical details including artery puncture and final recanalization times, and recanalization rates. Recanalization was graded by the modified thrombolysis in cerebral ischemia (mTICI) scale score (range 0: no flow beyond the occlusion, range 1: minimal reperfusion, range 2a: <50% of the affected endovascular territory reperfused, range 2b: >50% reperfusion, and range 3: complete reperfusion).^[18,19]^ Successful recanalization was defined as a mTICI score of 2b or 3. Hemorrhage complications on NCCT were classified according to the European Cooperative Acute Stroke Study 2 (ECASS-2) criteria as hemorrhagic infarction and parenchymal hematoma type 1 and type 2. Clinical outcome was quantified using the mRS at 3 months after EVT, the mRS is a 7-point scale ranging from 0 (no symptoms) to 6 (death). Unfavorable clinical outcome was defined as mRS scores of 3 or more at the 3-month follow-up.

### 2.5. Statistical analysis

Continuous variables were reported as the mean ± SD or median (interquartile range) for quantitative variables, as appropriate. Categorical variables were expressed as numbers (percentage). In the univariate analysis, independent sample *t* test or Mann–Whitney *U* test was used for the continuous variables, while the *χ*^2^ and Fisher exact tests were used to compare categorical variables. Multivariable regression analyses with 3 models were conducted. Model 1 adjusted for age and sex, Model 2 adjusted for all variables except baseline ASPECTS with at least marginal significance (*P* < .1) on univariate analysis, and Model 3 adjusted for all variables with at least marginal significance (*P* < .1) on univariate analysis. Receiver operating characteristic (ROC) curves were constructed by plotting test sensitivity against (1 – specificity). We used area under curve receiver operating characteristic (AUCROC) curve on NCCT, which were significantly associated with the outcome variable in univariable analysis, to assess the accuracy of NCCT to predict 3-month outcomes. Statistical analyses were performed with the SPSS package (IBM, Armonk, NY; Version 22.0) and MedCalc (Ostend, Belgium; Version 19.0). A value of *P* < .05 was considered statistically significant.

## 3. Results

### 3.1. Patient characteristics

Among the 235 patients with anterior circulation ischemic stroke who underwent EVT, 12 patients were excluded from this study (7 patients did not have available admission CT or follow-up CT, and 5 patients had incomplete outcome data), and 223 patients were included. Hyperdense lesions on NCCT immediately after EVT were observed in 191 (85.7%) patients, and 58.6% (112/191) of them had unfavorable outcomes at the 3-month followed-up; 32 (14.3%) patients had negative hyperdense, 15 (46.8%) patients had unfavorable outcomes, but none of them died. A total of 191 hyperdense lesion patients were included in the final analysis. There were 97 females (50.8%), and the mean age was 66.6 ± 8.3 years. The median admission NIHSS was 17 (interquartile range [IQR], 13–19). The median baseline ASPECTS was 8 (IQR, 7–9), and intravenous thrombolysis was administered in 70 (36.6%) patients. Successful recanalization on angiography (modified thrombolysis in cerebral infarction (mTICI) 2b/3) was achieved in 154 patients (80.6%), and the post-ASPECTS was 7 (IQR, 6–8). Symptomatic intracranial hemorrhage was observed in 18 patients (9.4%), and 10 patients were found to have PH2 on CT immediately post-thrombectomy, which we excluded from the hyperdense lesion group. The 3-month unfavorable outcomes were 58.6% (112/191) and the rate of mortality at 90 days was 22.9% (38/191). The demographics, baseline characteristics, treatments, and outcomes of the cohort are presented in Table 1.

**Table 1:** Demographics, baseline, and procedure-related variables between positive hyperdense lesion patients with favorable and unfavorable outcomes.

Characteristics	Overall (n = 191)	Favorable outcome (n = 79)	Unfavorable outcome (n = 112)	*P* value
Age, mean (SD), yr	66.6(8.3)	65.0(9.2)	67.7(7.4)	.030
Female sex, n (%)	97(50.8)	46(58.2)	51(45.5)	.084
Hypertension, n (%)	115(60.2)	44(55.7)	71(63.4)	.285
DM, n (%)	40(20.9)	15(19.0)	25(22.3)	.577
AF, n (%)	67(35.1)	21(26.6)	46(41.1)	.039
Smoking, n (%)	60(31.4)	21(26.6)	39(34.8)	.227
OAC, n (%)	8(4.2)	2(2.5)	6(5.4)	.475
DOACs, n (%)	3(1.6)	0(0)	3(2.7)	
VKA, n (%)	5(2.6)	2(2.5)	3(2.7)	
Baseline measurements				
SBP, median (IQR), mm Hg	149(130-158)	147(132-155)	153(129-164)	.129
Serum glucose, median (IQR), mmol/L	5.8(5.2-6.6)	5.6(5.3-6.6)	6.2(5.2-6.7)	.053
NIHSS score, median (IQR)	17(13-19)	15(12-18)	17(13-20)	.003
Baseline ASPECTS, median (IQR)	8(7-9)	8(7-9)	8(7-8)	.108
Occlusion site, n (%)				
ICA	61(31.9)	22(27.8)	39(34.8)	.309
M1	108(56.5)	47(59.5)	61(50.4)	.490
M2	22(11.5)	10(12.7)	12(10.7)	.679
Procedure process and results				
Intravenous thrombolysis, n (%)	70(36.6)	32(40.5)	38(33.9)	.938
ASITN/SIR ≥ 2, n (%)	81(42.4)	42(53.2)	39(34.8)	.012
mTICI, 2b or 3, n (%)	156(81.7)	75(94.9)	81(72.3)	<.001
Post-ASPECTS, median (IQR)	7(6-8)	8(7-8)	6(6-7)	<.001
Symptomatic intracranial hemorrhage, n (%)	18(9.4)	1(1.3)	17(15.2)	.001

AF = atrial fibrillation, ASITN/SIR = American Society of Interventional and Therapeutic Neuroradiology/Society of Interventional Radiology, ASPECTS = Alberta Stroke Program Early CT Score, DM = diabetes mellitus, DOACs = direct oral anticoagulants, ICA = internal cerebral artery, M1 = middle cerebral artery M1 segment, M2 = middle cerebral artery M2 segment, mTICI = modified Thrombolysis in Cerebral Ischemia, NIHSS = National Institute of Health Stroke Scale, OAC = oral anticoagulation, SBP = systolic blood pressure, VKA = vitamin K antagonist.

On univariate analysis (Table 2), the age of poor outcome patients was higher than of good outcome patients (67.7 vs 65.0; *P* = .030). Atrial fibrillation was higher more prevalent in patients with poor outcome than in patients with good outcome (41.6% vs 26.6%; *P* = .039), and patients with poor outcome had lower baseline NIHSS scores (median, 17 vs 15; *P* = .003). Poor outcome patients were had a lower post-ASPECTS than good outcome patients (median, 6 vs 8; *P* < .001). Good collateral circulation (American Society of Interventional and Therapeutic Neuroradiology/Society of Interventional Radiology ≥ 2) was less common in poor outcome patients than in good outcome patients (34.8% vs 53.2%; *P *= .012), as was symptomatic intracranial hemorrhage (1.3% vs 15.2%; *P *= .001). The successful reperfusion (mTICI > 2a) ratio in poor outcome patients was lower than that in good outcome patients (72.3% vs 94.9%; *P* < .001).

**Table 2:** Univariate analysis of predictors of 90 days unfavorable outcome after endovascular thrombectomy in positive hyperdense lesion patients.

Characteristics	OR	95% CI	*P* value
Age	1.040	1.003–1.077	.033
Female	1.667	0.932–2.987	.085
Hypertension	1.377	0.766–2.478	.285
DM	1.226	0.599–2.511	.577
AF	0.519	0.278–0.971	.040
Smoking	1.476	0.784–2.778	.228
SBP	1.009	0.996–1.023	.180
Serum glucose	1.131	1.017–1.258	.023
NIHSS	1.122	1.045–1.204	.001
Baseline ASPECTS	0.742	0.526–1.048	.090
Intravenous thrombolysis	0.801	0.423–1.517	.496
ASITN/SIR ≥ 2	0.471	0.261–0.848	.012
mTICI, 2b/3	0.386	0.171–0.872	.022
Post-ASPECTS	0.356	0.249–0.511	<.001
Symptomatic intracranial hemorrhage	13.958	1.817–107.227	.011

AF = atrial fibrillation, ASITN/SIR = American Society of Interventional and Therapeutic Neuroradiology/Society of Interventional Radiology, ASPECTS = Alberta Stroke Program Early CT Score, DM = diabetes mellitus, mTICI = modified Thrombolysis in Cerebral Ischemia, NIHSS = National Institute of Health Stroke Scale, SBP = systolic blood pressure.

**Figure 2.**
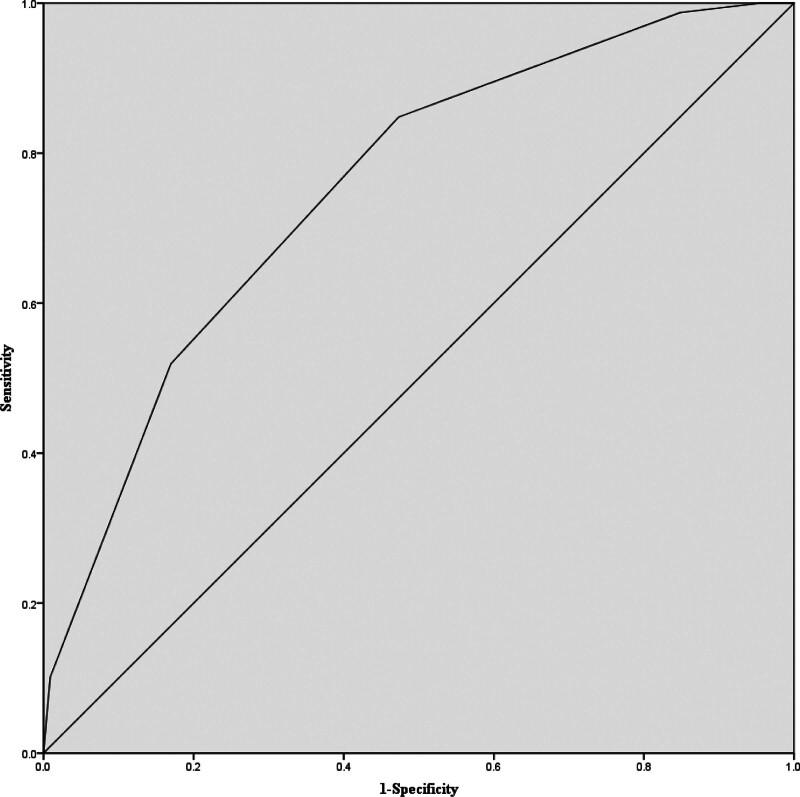
Receiver operating characteristic curve (ROC) for post-ASPECTS and poor functional outcome. The *x*-axis shows the 1-specificity, and the *y*-axis shows the sensitivity of post-ASPECTS for prediction of poor functional outcome. AUROC curve was 0.753 (95% CI 0.684–0.822).

Multivariate logistic regression analyses showed that post-ASPECTS was an independent predictor of unfavorable outcome after EVT in 3 models (Table 3). After adjusting for all variables with at least marginal significance (*P* < .1) on univariate analysis (Table 2), the odds ratio for poor outcome (mRS 3-6) was 0.390 (95% CI, 0.258-0.589; *P* < .001), with an AUCROC curve of 0.713 (95% CI, 0.646-0.780), while baseline NCCT-ASPECTS (OR 1.015; 95% CI, 0.719-1.434; *P *= .931) was not a predictor of outcome in our study. Through ROC analysis, we demonstrated that best cutoff point for post-ASPECTS to maximize the sensitivity and specificity when identifying patients with unfavorable outcomes was 7, the sensitivity was 0.830 (95 % CI, 0.748–0.895) and was specificity 0.519 (95% CI, 0.404–0.633), and the positive predictive value (PPV) and negative predictive value (NPV) was 71.0% (95% CI, 0.657-0.757) and 68.3 % (95% CI, 0.576-0.774), respectively. Figure 2 shows the AUCROC.

**Table 3:** Multivariable analysis of predictors of 90-day unfavorable clinical outcome in positive hyperdense lesion patients.

	Model 1			Model 2			Model 3		
	OR	CI	*P*	OR	CI	*P*	OR	CI	*P*
Female	1.238	0.639–2.397	.527	1.338	0.642–2.790	.437	1.310	0.627–2.736	.472
Age	1.029	0.988–1.071	.171	1.028	0.979–1.068	.312	1.026	0.981–1.072	.261
AF				0.493	0.230–1.055	.068	0.489	0.227–1. 051	.067
Serum glucose				1.108	0.984–1.247	.090	1.108	0.985–1.246	.088
NIHSS				1.148	1.038–1.269	.007	1.146	1.035–1.268	.009
Baseline ASPECTS							0.754	0.497–1.144	.185
ASITN/SIR ≥ 2				0.801	0.380–1.709	.876	0.744	0.344–1.610	.453
mTICI, 2b/3				0.461	0.161–1.322	.297	0.417	0.143–1.220	.110
Symptomatic intracranial hemorrhage				2.849	0.289–27.778	.092	2.475	0.249–24.39	.439
Post-ASPECTS	0.377	0.263–0.539	<.001	0.393	0.261–0.591	<.001	0.390	0.258–0.589	<.001
Female	1.238	0.639–2.397	.527	1.338	0.642–2.790	.437	1.310	0.627–2.736	.472
Age	1.029	0.988–1.071	.171	1.028	0.979–1.068	.312	1.026	0.981–1.072	.261
AF				0.493	0.230–1.055	.068	0.489	0.227–1.051	.067
Serum glucose				1.108	0.984–1.247	.090	1.108	0.985–1.246	.088
NIHSS				1.148	1.038–1.269	.007	1.146	1.035–1.268	.009
Baseline ASPECTS							0.754	0.497–1.144	.185
ASITN/SIR ≥ 2				0.801	0.380–1.709	.876	0.744	0.344–1.610	.453
mTICI, 2b/3				0.461	0.161–1.322	.297	0.417	0.143–1.220	.110
Symptomatic intracranial hemorrhage				2.849	0.289–27.778	.092	2.475	0.249–24.39	.439
Post-ASPECTS	0.377	0.263–0.539	<.001	0.393	0.261–0.591	<.001	0.390	0.258–0.589	<.001

Model 1 adjusted for age and sex. Model 2 adjusted all variables except baseline ASPECTS with at least marginal significance (*P* < .1) on univariate analysis. Model 3 adjusted for all variables with at least marginal significance (*P* < .1) on univariate analysis.

AF = atrial fibrillation, ASITN/SIR = American Society of Interventional and Therapeutic Neuroradiology/Society of Interventional Radiology, ASPECTS = Alberta Stroke Program Early CT Score, mTICI = modified Thrombolysis in Cerebral Ischemia, NIHSS = National Institute of Health Stroke Scale.

## 4. Discussion

In this retrospective study, the ASPECTS applied to hyperdense lesions on NCCT obtained immediately after EVT for acute anterior circulation large vessel occlusion predicted unfavorable outcome at 90 days with high specificity and NPV. Our study identified a score ≤ 7 as the best predictive value to forecast poor outcome in positive hyperdense lesion patients. Moreover, the post-ASPECTS seems to be more accurate than the baseline ASPECTS estimating 3-month prognosis.

Intracerebral hyperdense lesions on NCCT immediately performed in patients with AIS after intra-arterial thrombolysis were first reported in the early 1990s.^[20]^ The pathophysiology of hyperdense lesions associated with reperfusion therapies includes the disruption of the blood–brain barrier, direct vessel damage related to EVT, and toxicity secondary to thrombolytic drugs.^[21]^ Hyperdense lesions consist of contrast extravasation, intracerebral hemorrhage, or both.^[22]^ There are several definitions of high density, and we chose a definition of high density > 40 HU which is convenient for clinical use.^[16,17]^ In our study, we found that this definition has good sensitivity and specificity for predicting poor clinical outcomes. One CT is not enough for to distinguish between contrast extravasation and hemorrhage after EVT,^[23]^ and dual-energy CT and magnetic imaging resonance have already proven to be helpful in differentiating contrast extravasation and hemorrhage after EVT.^[24]^ However Renú et al^[21]^ found that both contrast extravasation and brain hemorrhage as classified by dual-energy CT were markers of poor outcome, even in patients selected by multimodal neuroimaging who achieved complete recanalization. They suggested that early blood-brain barrier disruption is a predictor of poor clinical outcomes. Although the hyperdense lesions should not necessarily be interpreted as hemorrhage alone, they were associated with clinical neurological function deterioration.^[25]^ The incidence of intracerebral hyperdense lesions shown on NCCT immediately after EVT varies greatly, ranging from 55% to 86%.^[8,21,23,24,26]^ The reason for such a wide variation may be due to the definition of hyperdense lesions, the number of patients, the rate of recanalization, the quality of the imaging equipment and the difference in the time interval between post-thrombectomy scans. Compared to previous studies,^[8,21,23,24,26]^ the rate of intracerebral hyperdense lesions in our study was relatively high (our study vs other studies, 83% vs 55%–86%). This finding may be because the f operating room is adjacent to the NCCT scan room in our center, The NCCT scan was performed immediately after EVT, and the interval from the post-thrombectomy to the NCCT scan was very short. Second, compared with intra-arterial thrombolysis, especially using retrievable stent, increases the direct damage to the endothelium of cerebral arteries when pulling thrombi, which may increase the incidence of hyperdense lesions in the brain. Third, it may be due to the relatively high vascular recanalization rate in our center.

The ASPECTS is a semi-quantitative scoring system for estimating early ischemic changes in patients with AIS in the anterior circulation during the acute phase by NCCT. It is widely used in the screening of EVT patients and has a certain ability to predict clinical outcome, but 1 of its disadvantages is its limited specificity, which restricts its predictive value. The conventional ASPECTS was not associated with good or poor outcome in AIS patients who undergo endovascular reperfusion.^[27,28]^ Our results were similar to those of previous studies. In our study, we did not find an association between the baseline NCCT ASPECTS and functional outcome (OR: 0.742; 95% CI: 0.526-1.048; *P* = .090). In addition to NCCT, ASPECTS is also widely used in evaluating the core volume of cerebral infarction by magnetic resonance imaging. ASPECTS has been applied to diffusion-weighted magnetic resonance imaging (DWI), which is much more sensitive and accurate in the detection of acute infarction than NCCT. A study showed that DWI-ASPECTS was superior to CT-ASPECTS in predicting functional outcome at 3 months.^[29]^ ASPECTS on CT angiography source images has been shown to be a more accurate predictor of final infarct volume and clinical outcome in AIS patients who receive EVT.^[30]^ The baseline ASPECTS applied to CT angiography source images strongly predicted futile recanalization and was a valuable tool for treatment decisions regarding the indication of revascularization therapies, and it was more powerful than NCCT in predicting the recanalization rate. In our study, the ASPECTS was applied to semi-quantitative evaluation of hyperdense lesions on NCCT immediately after EVT, and obtained a good predictive ability for poor clinical outcome. To the best of our knowledge, this is the first study focusing on ASPECTS applied to hyperdense lesions on NCCT immediately after thrombectomy in anterior circulation AIS and its predictive value for clinical outcome at 90 days. ASPECTS used on hyperdense lesions was easily and quickly applied in clinical practice, it might be helpful in identifying those patients with a higher risk of poor clinical outcome after EVT. It may not only provide prognostic information for clinicians and patients but also contribute to clinical management after EVT.

### 4.1. Limitations

Our study had several limitations: first, we did not distinguish between contrast extravasation and cerebral hemorrhage transformation in hyperdense lesions. Although some studies have shown that hemorrhage is more likely when the NCCT density is >90 HU,^[10]^ there is still a greater risk of missed diagnosis. Hemorrhage is often accompanied by contrast extravasation, and previous studies have shown that NCCT is not accurate enough to identify contrast extravasation or hemorrhage transformation after EVT.^[23]^ Dual-energy CT and magnetic resonance imaging can differentiate between contrast extravasation and hemorrhage, but these 2 examinations are not available in most developing country hospitals or cannot be easily performed immediately post-thrombectomy. After follow-up CT, we found that the prevalence of symptomatic cerebral hemorrhage was 9.4% (18/191). However, 10 of the 18 cases of symptomatic cerebral hemorrhage were found to be PH2 on CT immediately post-thrombectomy, which we excluded from the hyperdense lesion group. Only 4.2% (8/191) of patients with symptomatic intracerebral hemorrhage may be mixed with patients with hyperdense lesion. Considering the convenience, low cost, and specificity of NCCT, it seems to be an acceptable method to distinguish between contrast extravasation and intracerebral hemorrhage. This is why we believe our findings may be valid, despite this limitation. Second, this was a single-stroke-center, retrospective observational study. Patients were not systematically assessed, and there was a moderate number of cases, which could lead to selection bias.

In conclusion, the data from our study indicate that a post-ASPECTS ≤ 7 may indicate a high risk poor outcome at 3 months with a high specificity of −71.0% (95% CI, 0.657–0.757) and NPV of −68.3% (95% CI, 0.576–0.774), which might contribute to formulating management strategies in the acute stage. We suggest that when evaluating the effect of imaging markers on prognosis after EVT, post-thrombectomy ASPECTS needs to be considered as another baseline imaging factor. Further studies are needed to better estimate post-thrombectomy ASPECTS by dual-energy CT or magnetic resonance imaging.

## Author contributions

**Formal analysis:** Zhengzhou Yuan, Yuan Yang, Ying Luo.

**Methodology:** Jinglun Li, Hua Luo, Xiu Chen.

**Validation:** Renliang Meng, Yang Xie, Li Jiang.

**Writing – original draft:** Zhengzhou Yuan, Zhiyu Lv, Benbing Rong, Zuoxiao Li.

**Writing – review & editing:** Zhengzhou Yuan, Zuoxiao Li.
